# Docetaxel enhances lysosomal function through TFEB activation

**DOI:** 10.1038/s41419-018-0571-4

**Published:** 2018-05-23

**Authors:** Jianbin Zhang, Jigang Wang, Yin Kwan Wong, Xin Sun, Yun Chen, Liming Wang, Liu Yang, Liqin Lu, Han-Ming Shen, Dongsheng Huang

**Affiliations:** 1 Department of Oncology, Clinical Research Institute, Zhejiang Provincial People’s Hospital, People’s Hospital of Hangzhou Medical College, Hangzhou, China; 2 Department of Pharmocology, Yong Loo Lin School of Medicine, National University of Singapore, Singapore, 117600 Singapore; 3 Department of Physiology, Yong Loo Lin School of Medicine, National University of Singapore, Singapore, Singapore; 4 Department of Surgery, Key Laboratory of Tumor Molecular Diagnosis and Individualized Medicine of Zhejiang Province, Zhejiang Provincial People’s Hospital, Hangzhou, China; 5 Artemisinin Research Center, China Academy of Chinese Medical Sciences, Beijing, 100700 China

## Abstract

Docetaxel is an effective and commonly used chemotherapeutic drug for cancer. Autophagy has been reported to be involved in the anticancer mechanism of docetaxel. However, the effect of docetaxel on lysosomal function remains elusive. In the present study, we first found that docetaxel treatment enhances autophagic flux in different cancer cells. Moreover, docetaxel treatment activates lysosomal function and promotes its fusion with autophagosome. Second, doctaxel treatment activates TFEB (transcription factor EB), a key nuclear transcription factor in control of lysosome biogenesis and function. We found that docetaxel promotes TFEB nuclear translocation and increases its transcriptional activity while knockdown of TFEB impairs lysosomal activation by docetaxel. Thirdly, TFEB activation by docetaxel is mediated by ROS (reactive oxygen species) generation and scavenging of ROS suppresses TFEB activity and lysosomal function in docetaxel-treated cells. Finally, inhibition of lysosomal function leads to increased docetaxel-induced cell death, suggesting that lysosomal activation protects against docetaxel-mediated apoptosis. Taken together, our results provide novel insights into the regulatory mechanisms of docetaxel on lysosomes, which could facilitate the development of novel potential cancer therapeutic agents via lysosomal inhibition.

## Introduction

Gastric cancer, one of the most commonly occurring types of cancer, currently accounts for almost 10% of cancer-related deaths worldwide, making it the second most common cause of death due to cancer^1,2^. By the time of diagnosis, the majority of patients are already presenting metastasis with the cancer being unresectable. Palliative chemotherapy is the primary treatment prescribed for such surgically unfit patients^3^. In particular, fluoropyrimidines, platinum-containing agents such as cisplatin and taxanes, whether alone or in combination, are currently among the most effective and commonly used chemotherapy regimens^3,4^. Docetaxel is among the second generation of taxanes and demonstrates a stronger anticancer effect than paclitaxel, which has been widely applied in a variety of tumors, including advanced gastric cancer, non-small cell lung cancer, hormone-refractory prostate cancer and breast cancer^5–7^. It exerts its anticancer effect through inhibition of microtubule depolymerization, by promoting microtubule assembly and stabilizing microtubule structures. While docetaxel is among the more effective chemotherapeutic agents that are currently available, many obstacles remain in maximizing its anticancer efficacy in clinical application. For gastric cancers, the clinical response rate of docetaxel combination therapy with cisplatin or fluorouracil remains at an unsatisfactory 37%, with some patients reporting adverse effects with no benefit^5^. Thus, increasing the chemosensitivity to docetaxel has become a key area of focus for improving its therapeutic effects for patients with advanced gastric cancer.

Autophagy is a conserved process that selectively degrades cellular proteins and cytoplasmic organelles. It is implicated in many diseases, including neuronal degeneration diseases and cancer^8,9^. It has been reported^10,11^ that docetaxel induces autophagy in many cancer cells, such as human lung adenocarcinoma and prostate cancer. Mechanistic investigations have revealed that HMGB1 (high-mobility group box 1) promotes the formation of the Beclin1-PI3KIII complex via activation of the MEK (mitogen-activated protein kinase)-ERK (extracellular signal-regulated kinase) signaling pathway^10^, in turn regulating autophagosome formation. Further studies^10,12,13^ revealed that autophagy induction contributes to docetaxel resistance in some cancers and inhibition of autophagy can improve chemosensitivity to docetaxel and therapeutic index. Therefore, subsequent studies were performed to disrupt autophagy in order to enhance the antitumor efficacy of docetaxel through the co-delivery of autophagy inhibitors^12,14^. The chemotherapeutic potential of PEG-b-PLGA copolymer micelles combining docetaxel and the autophagy inhibitor CQ (chloroquine) has been investigated and the co-delivery micelles have displayed demonstrably superior therapeutic effects against cancer cells than either the free drug or docetaxel-loaded micelles^15^. This result provides a promising combination therapeutic strategy in enhancing the antitumor efficacy of docetaxel.

Lysosomes are acidic organelles containing many degradative enzymes, including proteases, nucleases, peptidases, phosphatases, lipases, glycosidases, and sulfatases. At the late stage of autophagy, autophagosome fuses with lysosome and the contents of the autophagosome are degraded by lysosomal enzymes^16,17^. Transcriptional factor EB (TFEB) is one of the most important molecular mechanisms regulating lysosomal function, which is downstream of mTOR (mammalian target of rapamycin)^9,18,19^. More recently, the lysosome has been revealed to participate in some anticancer drug resistance. In response to the sequestration of hydrophobic weak base drugs by lysosomes, lysosomal biogenesis (mediated by TFEB) takes place and results in enlarged lysosomal compartments which are then capable of further drug sequestration. Lysosomal sequestration of hydrophobic weak base chemotherapeutics such as sunitinib triggers TFEB-mediated lysosomal biogenesis, resulting in an enlarged lysosomal compartment which is then capable of further drug sequestration^20^. This reduces the accessibility of these drugs to their target sites and results in a markedly reduced cytotoxic effect. However, the role of lysosomal function in the anticancer effect of docetaxel is still unknown. Lysosomal inhibition could be a promising approach to improve chemosensitivity to docetaxel for anti-gastric cancer purposes.

In this study, we found that TFEB activation and associated autophagy and lysosomal levels increased rapidly in response to docetaxel treatment in gastric cancer cells. Knockdown of TFEB levels limited docetaxel-induced lysosomal activation, which enhanced the chemosensitivity of docetaxel and increased cancer cell apoptosis. Furthermore, our data verified a role for ROS production in the activation of TFEB by docetaxel. Taken together, our data support the notion that TFEB represents a suitable target in conventional chemotherapies of cancer.

## Results

### Docetaxel activates lysosomal function in different cancer cells

**Fig. 1:**
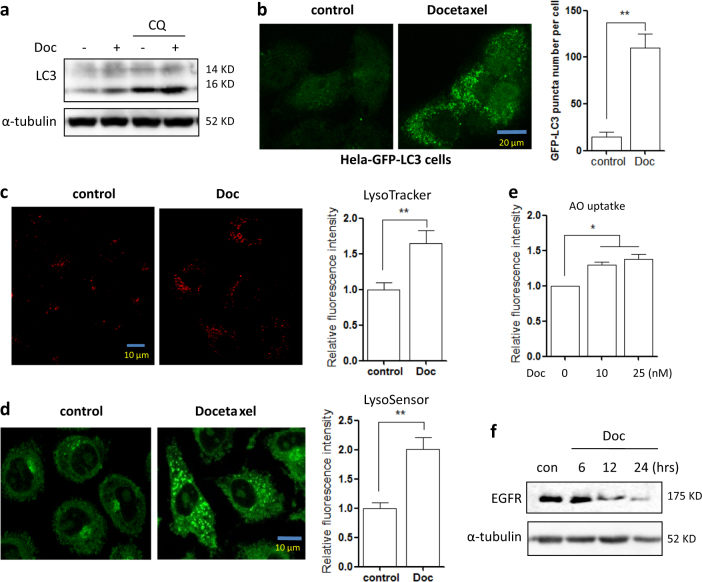
Docetaxel activates lysosomal function and induces autophagy in cancer cells. **a** AGS cells were treated with docetaxel (10 nM) with or without CQ (chloroquine) for 12 h. Cells were then harvested for western blotting to examine LC3-II levels. α-tubulin served as a loading control. **b** Hela cells stably expressing GFP-LC3 were treated with docetaxel (10 nM, 12 h) and then cells were examined by confocal microscopy (scale bar 20 μm).The number of GFP-LC3 puncta was quantified (right panel). ** *P* *<* 0.01 **c**, **d** AGS cells were treated with docetaxel (10 nM) for 12 h followed by loading with LysoTracker Red DND-99 (50 nM) or LysoSensor Green DND-189 (1 μM) for 15 min. Fluorescence intensity was measured under confocal microscope (scale bar 10 μm) and quantified using flow cytometry (right panel). **P *<* 0.01 (**e**) AGS cells were treated with docetaxel (10 or 25 nM) for 12 h. AO staining was performed and analyzed using flow cytometry. The numeric data are presented as means ± SD from three independent experiments. Student’s *t* test, *P *<* 0.05, **P *<* 0.01. **f** AGS cells were treated with docetaxel (10 nM) for different time points (6, 12, or 24 h) and western blotting was performed to detect EGFR protein level. α-tubulin was used as loading control

We first sought to examine the effect of docetaxel treatment on the autophagy process. In the human gastric cancer cell line AGS, docetaxel treatment led to an increase in the levels of the commonly used autophagy marker LC3-II (Fig. 1a). In docetaxel-treated Hela cells, the number of GFP-LC3 puncta was also significantly increased (Fig. 1b). We then determined changes in autophagy flux via the addition of the lysosomal inhibitor CQ (chloroquine), observing a further increase in LC3-II levels by docetaxel (Fig. 1a). It indicates that autophagy flux is also increased.

To examine the effect of docetaxel on lysosome, several assays were employed to determine the changes in lysosomal function in different cells. Firstly, as shown in Fig. 1c, d, in docetaxel-treated AGS cells, cell fluorescence under LysoTracker and LysoSensor staining was significantly increased, suggesting that docetaxel treatment enhanced lysosomal acidification. In another human gastric cancer cell BGC803, LysoTracker staining showed that docetaxel significantly increased cell fluorescence (SF. 1a). This was further confirmed by acridine orange (AO) staining, an orange/red fluorescent chelating dye that accumulates in acidic organelles, which showed an increase in red signal (Fig. 1e). Secondly, the Magic Red^TM^ assay for cathepsin was performed to measure the enzymatic activities of lysosomal cathepsin B and L, where a 1.5-fold increase of cell fluorescence intensity was observed following 12 h of docetaxel treatment in AGS cells (SF. 1b and 1c). Lastly, we measured changes in EGFR (epidermal growth factor receptor) levels, as EGFR is a known target of degradation by lysosomes. As shown in Fig. 1f, docetaxel induced a time-dependent degradation of EGFR in AGS cells, further indicating an enhancement in lysosomal degradative functions.

### Docetaxel promotes the fusion of autophagosome and lysosome in different cancer cells

**Fig. 2:**
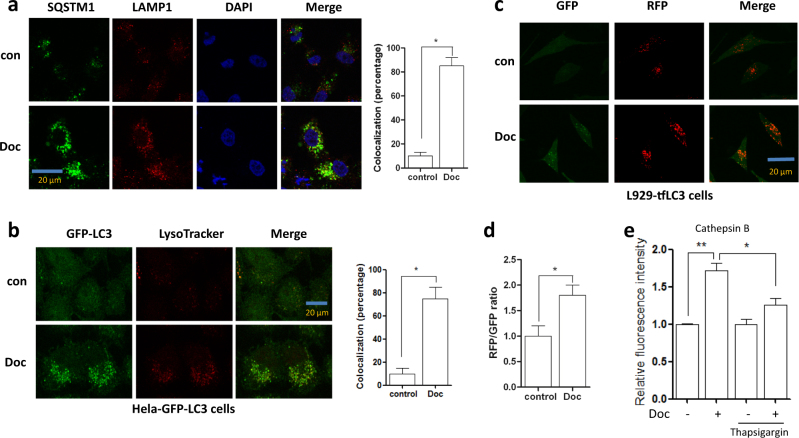
Docetaxel promotes the fusion of autophagosome and lysosome in different cancer cells. **a** AGS cells were treated with docetaxel (10 nM) for 12 h. After immunostaining with SQSTM1 (Alexa Fluor 488, green) and LAMP1 (Alexa Fluor 594, red), cells were examined by fluorescence microscopy (scale bar 20 μm). The colocalization of SQSTM1and LAMP1 was calculated and statistically analyzed (right panel). *P *<* 0.05. **b** Hela cells stably expressing GFP-LC3 were treated with docetaxel (10 nM, 12 h). After staining with LysoTracker Red, cells were examined by confocal microscopy (scale bar 20 μm).The colocalization of GFP-LC3 puncta (green) and lysosome (red) was calculated and statistically analyzed (right panel). *P *<* 0.05. **c**, **d** Docetaxel increased the RFP signal in the L929-tfLC3 cells. Cells were treated with docetaxel (10 nM) for 12 h and then cells were examined under confocal microscope (scale bar 20 μm). The ratio of RFP to GFP was calculated and statistically analyzed. *P *<* 0.05. **e** AGS cells were treated with docetaxel (10 nM, 12 h) in the presence or absence of thapsigargin (100 nM). After loading with Magic Red^TM^ for cathepsin B, cell fluorescence was measured by flow cytometry. *P *<* 0.05; **P *<* 0.01

During the late stages of autophagy, autophagosome fuses with lysosome and is subsequently degraded^21^. It has been proved that this fusion is required for lysosomal activation^21^. Here, we also determined the effect of docetaxel on the fusion of autophagosome and lysosome. As shown in Fig. 2a, in docetaxel-treated AGS cells, there was a significant increase of colocalization of SQSTM1 (the known autophagy substrate) and LAMP1, indicating the enhancement of autophagosome−lysosome fusion. In Hela cells, docetaxel treatment significantly increased the colocalization of GFP-LC3 puncta (autophagosome marker) and LAMP1 (Fig. 2b). Moreover, we used L929 cells stably expressing tfLC3B (mRFP-GFP tandem fluorescent‑tagged LC3B) to examine autophagosome−lysosome fusions. In this RFP−GFP tandem construct, the RFP component is stable under the acidic environment of the lysosomes while GFP is degraded. RFP-positive/GFP-negative puncta can thus be used as an indicator of autophagosome−lysosome fusion. As shown in Fig.  2c, d, we observed a significant increase in RFP-only puncta following docetaxel pretreatment, suggesting that docetaxel enhances autophagy by promoting autophagosome−lysosome fusion. Finally, we treated cells with thasigargin, a chemical commonly used to block the fusion of autophagosome and lysosome^22^. The Magic Red for cathepsin B staining showed a significant decrease of cell fluorescence in docetaxel-treated cells when adding thapsigargin (Fig. 2e), indicating that the enhancement of autophagosome−lysosome fusion by docetaxel is important for lysosomal activation.

### Docetaxel increases TFEB transcriptional activity in AGS cells

**Fig. 3:**
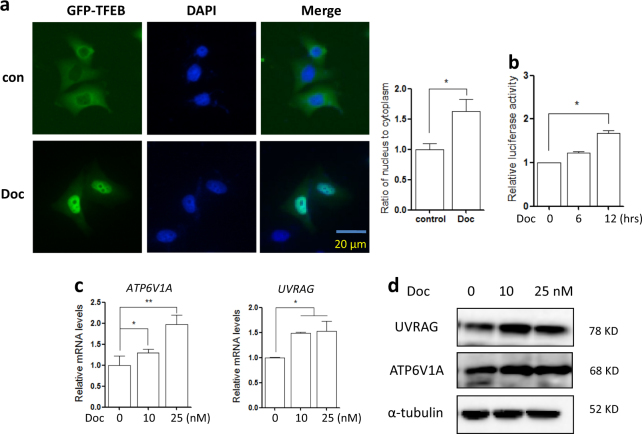
Docetaxel increases TFEB transcriptional activity. **a** Docetaxel treatment enhanced TFEB nuclear translocation (10 nM; 12 h). Live-cell imaging of GFP-TFEB (green) and DAPI (blue) in AGS cells showed an enrichment of the GFP-TFEB signal in the nucleus (scale bar 20 μm). The ratio of nuclear to cytosolic TFEB was calculated and statistically analyzed. *P *<* 0.05. **b** The TFEB-luciferase reporter constructs were first transiently transfected into AGS cells and then cells were treated with docetaxel (10 nM) for 12 h. The relative luciferase activity was measured using Dual-Luciferase^®^ Reporter assay. RLU stands for relative luciferase units. Error bars represent the standard deviation from two independent experiments. **c** AGS cells were treated with docetaxel (10 nM; 12 h) followed by cell harvesting and RNA extraction. Changes in mRNA levels of some known TFEB target genes were measured using real-time PCR. GAPDH served as a loading control. All values are means ± SD at least three independent experiments. Student’s *t* test, *P *<* 0.05; **P < 0.01. **d** As in (**c**), cells were harvested for western blotting to detect the ATP6V1A and UVRAG levels. α-tubulin was used as loading control

TFEB (transcription factor EB) regulates the expression of autophagy and lysosomal-related genes and serves as a master regulator for lysosome biogenesis^9^. Here, we measured the transcriptional activity of TFEB in response to docetaxel treatment. Firstly, we determined the localization of TFEB in docetaxel-treated cells. In AGS cells transiently expressing GFP-TFEB, fluorescence microscope analysis showed that docetaxel treatment promotes the translocation of TFEB into the nucleus (Fig. 3a). Secondly, a TFEB promoter-driven luciferase reporter was used to measure the transcriptional activity of TFEB. As shown in Fig. 3b, docetaxel significantly increased the luciferase activity of TFEB at 12 h point. Finally, we measured the levels of several known targets of TFEB, namely ATP6V1A (V-type proton ATPase catalytic subunit A) and UVRAG (UV radiation resistance-associated gene). In AGS cells, both mRNA and protein levels of these two targets were significantly upregulated following docetaxel treatment (Fig. 3c, d). Taken together, these results suggest that docetaxel enhances the transcriptional activity of TFEB.

### Knockdown of TFEB impairs lysosomal activation and its fusion with autophagosome by docetaxel

**Fig. 4:**
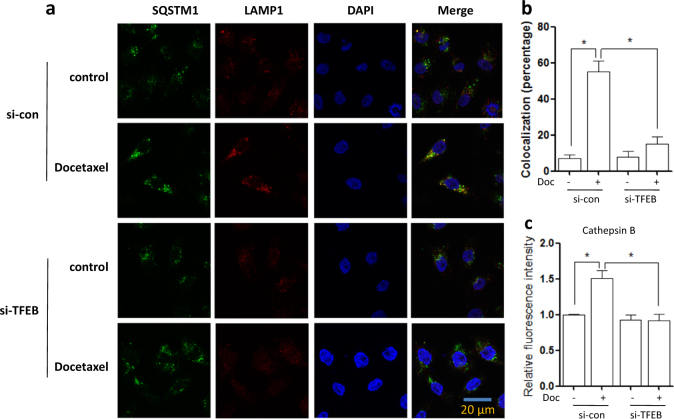
Knockdown of TFEB impairs lysosomal activation by docetaxel. **a**, **b** AGS cells were transfected with scrambled or TFEB siRNA for 48 h and then treated with docetaxel (10 nM) for 12 h. After immunostaining with SQSTM1 (Alexa Fluor 488, green) and LAMP1 (Alexa Fluor 594, red), cells were examined by fluorescence microscopy (scale bar 20 μm). The colocalization of SQSTM1 and LAMP1 was calculated and statistically analyzed. *P *<* 0.05. **c** As in (**a**), at the indicated time, cells were loaded with Magic Red cathepsin B for 15 min. Fluorescence intensity of 10,000 cells per sample was measured by flow cytometry. *P *<* 0.05

We next carried out a transient TFEB knockdown in order to validate the regulatory role of TFEB in lysosomal function. As shown in Fig. 4a, b, we determined the effect of TFEB on autophagosome−lysosome fusion. As expected, under TFEB knockdown, the colocalization of SQSTM1 and LAMP1 was significantly decreased in docetaxel-treated AGS cells. Meanwhile, TFEB knockdown also attenuated the docetaxel-induced increase of cathepsin B activity (Fig. 4c). Our results suggest that TFEB activation is integral to the changes in lysosomal activation following doctaxel treatment. All these results indicate that TFEB activity is required for docetaxel-induced lysosomal activation.

### Docetaxel activates TFEB function through ROS generation

**Fig. 5:**
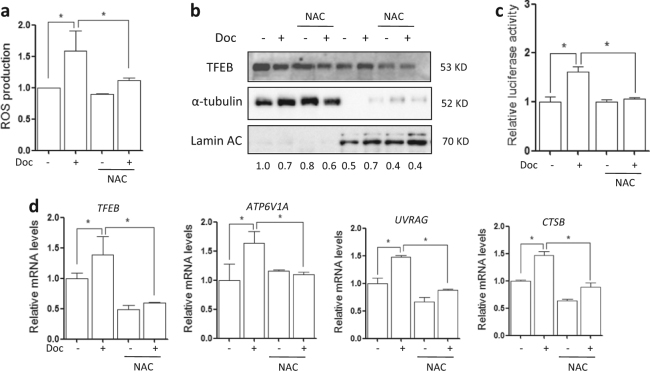
Docetaxel enhances TFEB activity through ROS generation. **a** AGS cells were treated with docetaxel (10 nM) with or without NAC (5 mM) for 12 h followed by incubation with CM-H2DCFDA. The cells’ fluorescence intensity was then measured using flow cytometry. *P *<* 0.05. **b** As described in (**a**), nuclear and cytosolic fractions from both control and docetaxel-treated cells were probed for TFEB and then quantified. The same membrane was then stripped and reprobed for α-tubulin or Lamin AC as loading control. **c** AGS cells were transiently transfected with the TFEB-luc reporter construct. After 24 h, the cells were treated with docetaxel (10 nM) for another 12 h with or without NAC (5 mM) and the relative luciferase activity was measured. RLU refers to relative luciferase units. *P *<* 0.05. **d** As in (**a**), after treatment, AGS cells were harvested for RNA extraction. Real-time PCR was used to determine mRNA level changes in known TFEB target genes. GAPDH served as a loading control. All values are means ± SD at least three independent experiments. Student’s *t* test, *P *<* 0.05

To elucidate the link between docetaxel treatment and TFEB activity, we measured the changes in ROS (reactive oxygen species) levels in docetaxel-treated cells using the CM-H2DCFDA probe^23^. As shown in Fig. 5a, a significant increase of green fluorescence representing ROS production was observed after docetaxel treatment. *N*-acetyl cysteine (NAC) is a cell-penetrating antioxidant that replenishes intracellular GSH (glutathione) so as to protect the cells from oxidative stress. The addition of NAC totally blocked the ROS generation by docetaxel (Fig. 5a). Therefore, docetaxel induces ROS generation in AGS cells.

TFEB is a key regulator of autophagy-lysosome pathway to promote protein clearance^9^. Normally, TFEB is located in the cytosol and on the lysosomal surface where it interacts with mTOR (mammalian target of rapamycin)^18^. Based on the fact that ROS is involved in the TFEB nuclear translocation^23^, we then tested whether docetaxel activates TFEB via increasing ROS production. To do this, we treated AGS cells with docetaxel in the presence of NAC. As shown in Fig. 5b and SF. 2, NAC treatment abolished docetaxel-induced TFEB nuclear translocation. Moreover, we determined the effect of ROS production on TFEB transcriptional activity. As described previously^24^, we transiently transfected AGS cells with a TFEB promoter-driven luciferase reporter construct, before subjecting the cells to docetaxel and NAC co-treatment. As shown in Fig. 5c, docetaxel significantly increased the luciferase activity of TFEB but it was abolished by NAC. At the same time, NAC treatment also abolished the upregulation of TFEB target genes described previously, including ATP6V1A, UVRAG, and CTSB. Taken together, our results suggest that docetaxel-induced enhancement of TFEB transcriptional activity is mediated, at least in part, through ROS generation.

In addition, we also determined lysosomal function in docetaxel-treated AGS cells when scavenging ROS. As shown in SF. 3a, LysoTracker staining showed that docetaxel enhanced acidification of lysosome under microscope but it was impaired when adding NAC. Similarly, NAC treatment weakened lysosomal cathepsin B and L activities in docetaxel-treated cells (SF. 3b and 3c), as well as the degradation of EGFR (SF. 3d). In SF. 4, when scavenging ROS, the enhanced colocalizaton of GFP-LC3 and LAMP1 was also disrupted in docetaxel-treated Hela cells. The above results demonstrate that ROS production is required for docetaxel-caused lysosomal activation.

### Lysosomal activation protects from cell apoptosis by docetaxel

**Fig. 6:**
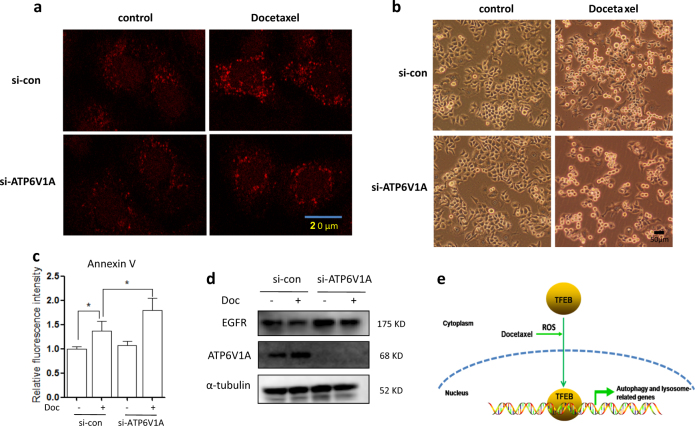
Lysosomal inhibition sensitizes docetaxel-induced cell death. **a** AGS cells were transfected with scrambled or ATP6V1A siRNA for 48 h and then treated with docetaxel (10 nM; 12 h). After loading with LysoTracer Red, cells were examined under confocal microscope. Scale bar 20 μm. **b**, **c** As in (**a**), cells were treated with docetaxel (25 nM) for 24 h. Changes in AGS cell morphology following each treatment were examined and captured with an inverted microscope (scale bar 50 μm). Cell pellets were subsequently collected and quantification of cell death was performed using Annexin V staining. Statistical significance (*P *<* 0.05) is indicated in the bar chart. **d** As in (**b**), after treatment, cells were harvested and western blotting was performed to detect ATP6V1A and EGFR levels. α-tubulin was used as loading control. **e** The schematic model of the enhanced function of lysosome by docetaxel

Finally, we sought to examine the functional role of lysosome activation in docetaxel-induced cytotoxicity. ATP6V1A is a component of vacuolar ATPase (V-ATPase), a multisubunit enzyme that mediates acidification of lysosome^25^. We first knocked down ATP6V1A expression in AGS cells and then treated cells with docetaxel. As shown in Fig. 6a and SF. 5a, LysoTracker staining showed that knockdown of ATP6V1A impaired the lysosomal activation by docetaxel in AGS cells. Changes in cell morphology indicated that docetaxel treatment led to increased cell death in ATP6V1A knockdown AGS cells (Fig. 6b). Next, we examined the type of cell death using Annexin V staining, which binds to phosphatidylserine on the outer leaflet of the plasma membrane and is commonly used to detect apoptotic cells. Annexin V staining followed by flow cytometry was used to quantify cell apoptosis in docetaxel-treated AGS cells. As shown in Fig. 6c, docetaxel treatment significantly increased cell fluorescence and knockdown of ATP6V1A led to a further increase in cell fluorescence under docetaxel treatment. In SF. 5b and 5c, in docetaxel-treated AGS cells, knockdown of TFEB led to a significant increase of cell apoptosis. In addition, in ATP6V1A knockdown AGS cells, the degradation of EGFR by docetaxel was impaired, indicating the lysosomal inhibition (Fig. 6d). The above results suggest that lysosomal inhibition could possibly sensitize cells towards docetaxel-induced apoptosis. These results support a cytoprotective role of lysosomal activation under docetaxel treatment.

## Materials and methods

### Reagents and antibodies

The antibodies used in our experiments included: ATP6V1A (Abcam, ab199326), EGF receptor (Cell Signaling Technology, 4267), LC3 (microtubule-associated protein 1 light chain 3) (Sigma, L7543), α-tubulin (Sigma, T6199), Lamin AC (Cell Signaling Technology, 2032), LAMP1 (Cell Signaling Technology, 9091), SQSTM1 (Sigma, SAB1406748), TFEB (Bethyl Laboratories, A303-673A).

The chemicals used in our experiments were: Annexin V Pacific Blue™ conjugate (Thermo Fisher Scientific, A35122), acridine orange (AO) (Immunochemistry Technologies, LLC, 6130), docetaxel (Sigma, 01885), chloroquine (CQ) (PubChem, 2719), lysoTracker Red DND-99 (Invitrogen, L7528), lysoSensor green DND-189 (Invitrogen, L7535), Magic Red^TM^ cathepsin B and L reagent with Acridine Orange (Immunochemistry Technologies, LLC, 937/938/6130), thapsigargin (Sigma, T9033).

### Cell culture

AGS and BGC803 cells were obtained from ATCC (AmericanType Culture Collection). Hela cells stably expressing GFP-LC3 were kindly provided by Dr. N. Mizushima (Tokyo Medical and Dental University, Japan). The tfLC3 stably transfected L929 cells were from Prof. Han-Ming Shen's lab (National University of Singapore, Singapore). Cells were maintained in DMEM (Sigma, D1152) containing 10% fetal bovine serum (HyClone, SV30160.03) in a 5% CO_2_ atmosphere at 37 °C.

### Acridine orange (AO) staining

AO is a lysosomotropic weak base and a concentration-dependent meta-chromatic fluorophore. Cells were stained with AO at a concentration of 5 μg/ml for 15 min and then washed with PBS. Fluorescence intensities of 10,000 cells per sample were measured by flow cytometry using the FACS cytometer (BD Biosciences).

### Estimation of intralysosomal pH using lysoTracker

The intralysosomal pH was estimated using LysoTracker, following the manufacturer’s instructions. The fluorescence intensity was observed under fluorescence microscope (Nikon ECLIPSE) and representative cells were selected and photographed.

### Cathepsin B and L activity assay

Following an earlier report^1^, cells were cultured in 12-well plates and treated as indicated. After treatments, cells were further loaded with Magic Red cathepsin B or L reagent for 15 min. Fluorescence intensities of 10,000 cells per sample were measured by flow cytometry using the FACS cytometer (BD Biosciences).

### Measurement of cellular fluorescence using fluorescence microscopy

Cells were seeded onto glass slides. After the designated treatments, cells were first fixed with 4% paraformaldehyde and permeabilized by 0.25% Triton X-100. And then cells were incubated with SQSTM1 or LAMP1 antibodies, respectively. After incubation in fluorochrome-conjugated secondary antibody, cells fluorescence intensity was observed under fluorescence microscope (Nikon ECLIPSE) and representative cells were selected and photographed.

### Luciferase assay

As described before^23^, the luciferase vector was transiently transfected into AGS cells using Lipofectamine 2000 transfection reagent (Invitrogen, 11668) according to the manufacturer’s protocols. After the designated treatments, the luciferase activity was measured using a Dual-Luciferase Reporter Assay System (Promega, E1960) based on the protocol provided by the manufacturer.

### Cell fractions preparation

AGS cells were treated with docetaxel at different time points. After that, nuclear and cytosolic extracts were then prepared with NE-PER^®^ nuclear and cytoplasmic extraction reagents (Pierce, 78833) according to the manufacturer’s protocol.

### Small interfering RNA (siRNA) and transient transfection

The scrambled RNAi oligonucleotides and siRNAs targeting TFEB (Dharmacon, 21425) or ATP6V1A (GenePharma, Shanghai) were transfected into AGS cells using the DharmaFECT 4 Transfection Reagent (Dharmacon, T-2001-02) according to the manufacturer’s protocol.

### Western blotting

Cells were lysed in Laemmli SDS buffer (62.5 mM Tris, pH6.8, 25% glycerol, 2% SDS, phosphatase inhibitor and proteinase inhibitor cocktail). An equal amount of protein was resolved by SDS-PAGE and transferred onto PVDF membrane. After blocking with 5% nonfat milk, the membrane was probed with designated primary and secondary antibodies, developed with the enhanced chemiluminescence method and visualized with the ChemiDoc MP (Bio-Rad).

### Reverse transcription and quantitative real-time PCR

RNA was extracted with the RNeasy kit (Qiagen, 217004). One microgram of total RNA was used for a reverse transcription reaction using High-Capacity cDNA Reverse Transcription kit (Applied Biosystems, 4368814). Real-time PCR was performed to measure the mRNA expression levels using SsoFast Eva Green Supermix (Bio-Rad, 172-5201) and CFX96 Touch Real-time PCR Detection System (Bio-Rad). Glyceraldehyde-3-phosphatedehydrogenase (GAPDH) was used as an internal control of RNA integrity. Real-time PCR was performed in triplicate. The primers used for ATP6V1A, TFEB, UVRAG, and GAPDH were based on the previous report^21^.

### Detection of viable and dead cells

Cell death was determined quantitatively and qualitatively using the following two assays, which are (i) morphological changes under phase-contrast microscopy; (ii) Annexin V staining coupled with flow cytometry. For Annexin V staining, the medium in each well was collected and cells were harvested with trypsin after treatments. Then, cell pellets obtained were resuspended in 1× Binding buffer containing 5 μl of Annexin V and incubated for 15 min at room temperature. Ten thousand cells from each sample were analyzed with FACS Calibur flow cytometry (BD Bioscience, San Jose, CA) using CellQuest software.

### Statistical analysis

All western blotting and image data presented are representatives from at least three independent experiments. The numeric data are presented as means ± SD from 2 to 3 independent experiments (each in duplicates or triplicates) and analyzed using Student’s *t* test.

## Discussion

In the present study, we sought to investigate the relationship between lysosomal function and the chemotherapeutic response to docetaxel in cancer cells and further explore its possible mechanism, trying to provide a support to chemotherapy choice for cancer patients in clinical practice. Our findings demonstrate that lysosomal inhibition can enhance chemosensitivity to docetaxel in cancer.

Docetaxel is a prominent and important chemotherapeutic drug that has been successfully employed in patients with various malignancies. However, its efficacy has been considerably limited by multidrug resistance^13,26^. It is known^10,11^ that docetaxel treatment substantially upregulates autophagy within tumors as indicated by changes in well-described autophagy markers including an increase in LC3 levels and a corresponding decrease in SQSTM1/p62. As autophagy is able to promote cell survival by recycling cellular components in cells damaged by chemotherapy^8,27^, it is reasonable to suggest that autophagy may also contribute to docetaxel chemoresistance. Indeed, co-delivery of docetaxel and autophagy inhibitor CQ can overcome docetaxel resistance^12,15^. However, the role of lysosomal function in the chemotherapeutic function of docetaxel remains poorly understood. In different cancer cells, we observed that lysosomal functions are stimulated by docetaxel treatment, as evidenced by (i) an increase in lysosomal acidification (reduced lysosomal PH) (Fig. 1c−e and SF. 1a), (ii) enhanced lysosomal cathepsin B activity (SF. 1b and 1c); and (iii) increased lysosomal degradative activity with EGFR degradation (Fig. 1f). It has been shown^20^ that lysosomal sequestration of various chemotherapeutic agents leads to reduced accessibility of such agents to their target sites, which results in a significant reduction in cytotoxicity that underlies drug resistance. We thus speculate that lysosomal activation is associated with chemosensitivity to docetaxel. Indeed, when lysosomes are inhibited by TFEB knockdown, docetaxel treatment led to increased cancer cell death (Fig. 6 and SF. 5). Our results support the notion that docetaxel and lysosomal inhibition can function as a combined therapeutic strategy for enhanced antitumor therapy.

ROS are naturally produced as by-products during oxygen metabolism and play a vital role in cellular homeostasis^28^. Apart from such endogenous sources, ROS levels can also increase as a response to stress and external stimuli including UV, heat exposure, and chemical stimulation. ROS are known to play important roles in various physiological and pathological processes such as autophagy and cell death^29,30^. In our study, we further observed that ROS production by docetaxel is associated with lysosomal activation. As shown in Fig. 5a, docetaxel treatment led to the increase of ROS levels in AGS cells and the addition of NAC abolished the generation of ROS. Accompanied by the increase of ROS level, lysosomal function was enhanced by docetaxel (SF. 3). In docetaxel-treated cells, lysoTracker staining showed higher fluorescence intensity and magic red staining showed enhanced lysosomal enzyme activity (Fig. 1 and SF. 1). In the presence of NAC, the enhancement of lysosomal activation and its fusion with autophagosome by docetaxel was impaired in AGS cells (SF. 3 and 4). This indicates that ROS production is required for lysosomal activation and autophagy induction by docetaxel.

TFEB is the master regulator of lysosome biogenesis through transcriptional regulation of its target genes closely related to lysosomal structure and function^9,19^, including hydrolases, lysosomal membrane proteins, and the V-ATPase complex. In our study, docetaxel activated lysosomal function via the enhancement of TFEB activity, including the promotion of its nuclear translocation, the increase of its luciferase activity, and upregulation of its target genes (Fig. 3). When TFEB is knocked down, lysosomal activation was impaired in docetaxel-treated cells (Fig. 4). In response to stress conditions, cellular accumulation of ROS activates transcription factors^23,30^, such as P53, HIF-1, Nuclear factor-like 2(NRF2), Forkhead box O3 (FOXO3), and TFEB, which upregulate the transcription of several genes involved in autophagy. As expected, our findings also showed docetaxel-induced TFEB activation is dependent on the production of ROS. Following treatment with ROS scavengers, docetaxel failed to activate TFEB function in AGS cells (Fig. 5), accompanied by the blockage of TFEB nuclear translocation and the abolishment of upregulation in its target genes.

Taken together, our findings demonstrated the novel efficacy of the anticancer drug docetaxel in gastric cancer by increasing lysosomal function to protect from cell apoptosis via enhancing TFEB activity (Fig. 6e). Thus, the inhibition of lysosomal function can be developed as a novel method to increase chemosensitivity to docetaxel.

## Electronic supplementary material

supplementary Figure 1

supplementary Figure 2

supplementary Figure 3

supplementary Figure 4

supplementary Figure 5

supplementary materials

Supplementary figure legends
