# Supplementary figures and images for: Docetaxel enhances lysosomal function through TFEB activation
Source: Cell Death Dis. 2018 May 23;9(6):614. doi: 10.1038/s41419-018-0571-4 (PMC5966422; doi:10.1038/s41419-018-0571-4)

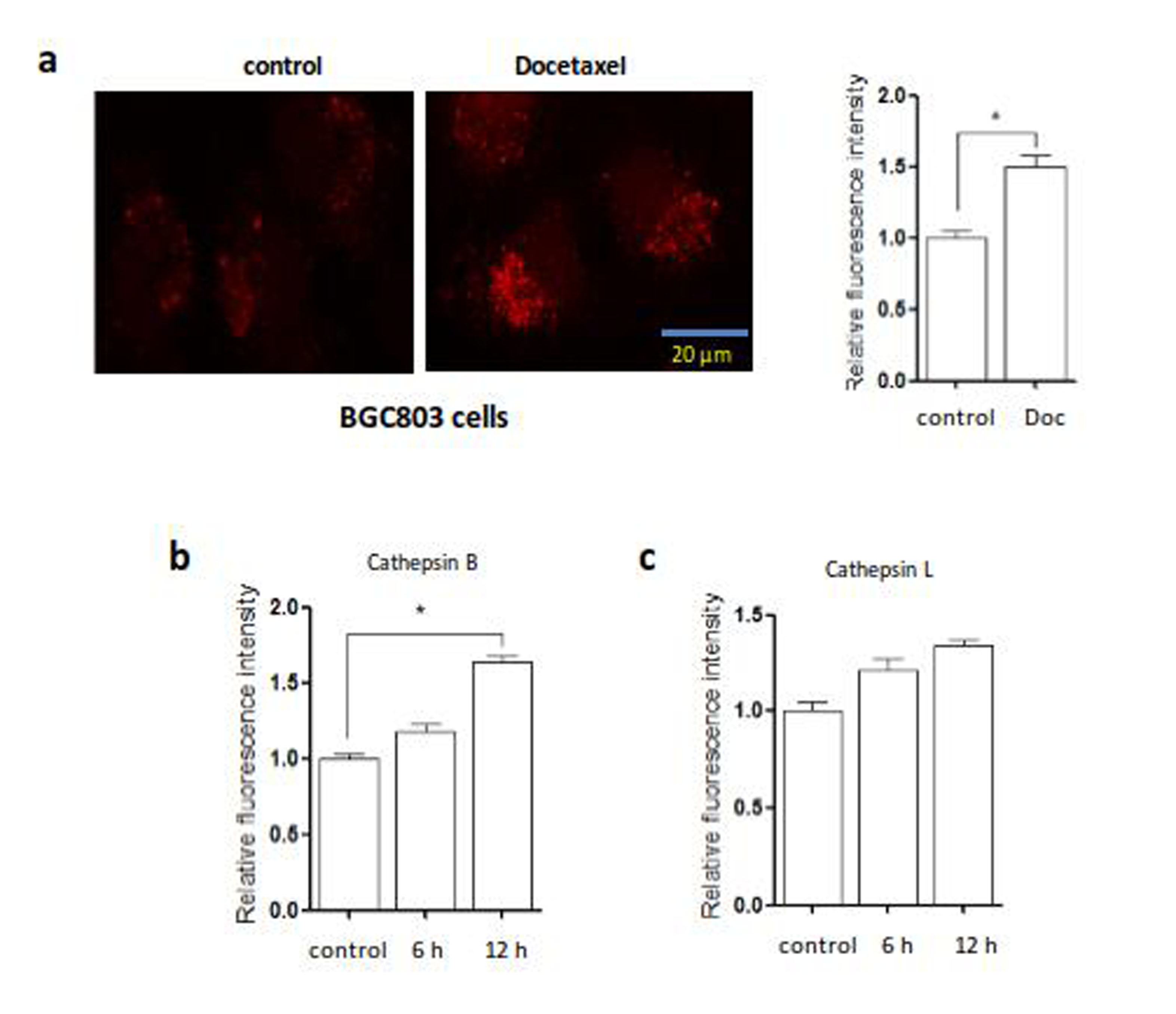

Supplement: Supplementary file 1 — supplementary Figure 1 [file 41419_2018_571_MOESM1_ESM.jpg]

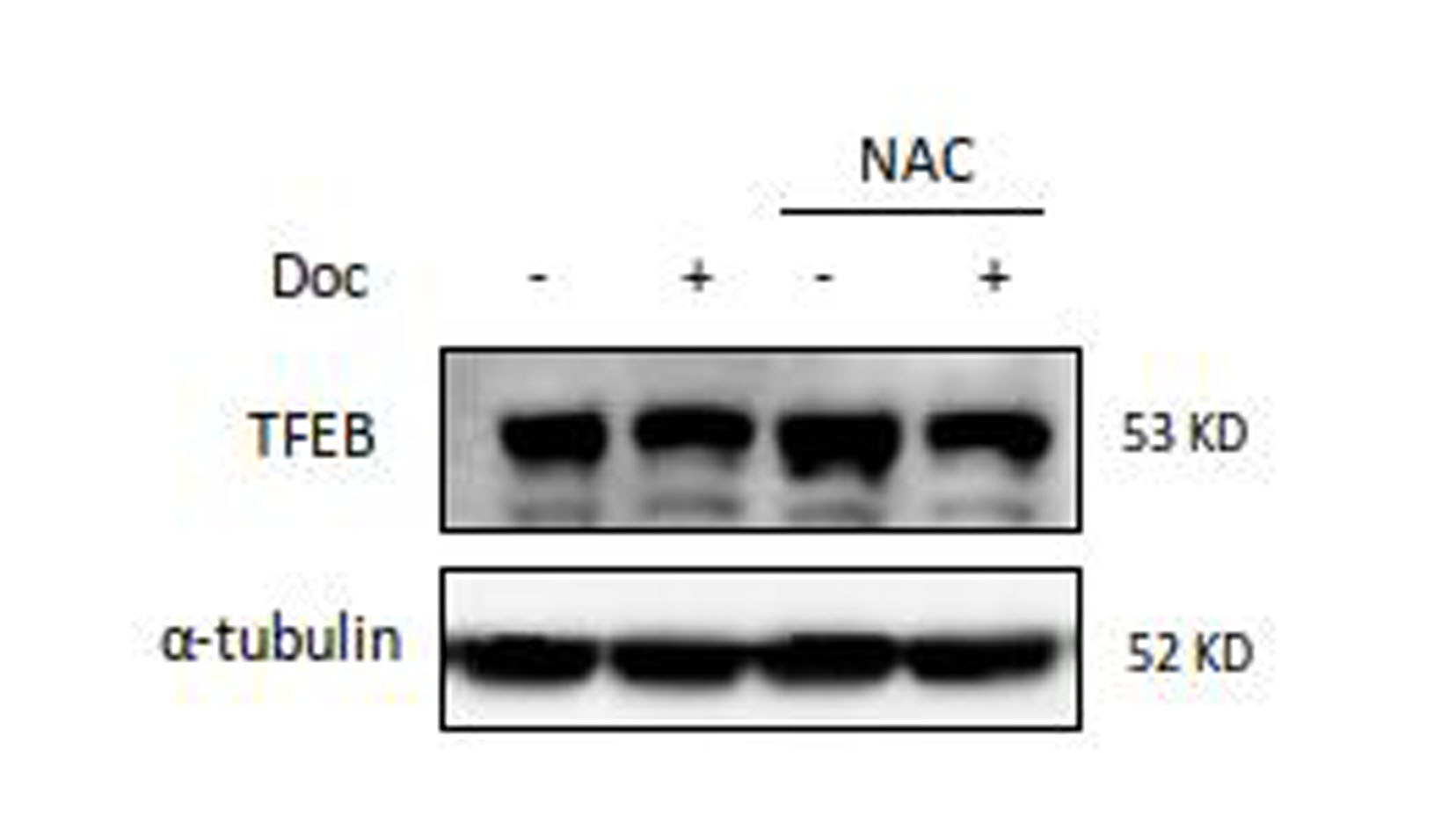

Supplement: Supplementary file 2 — supplementary Figure 2 [file 41419_2018_571_MOESM2_ESM.jpg]

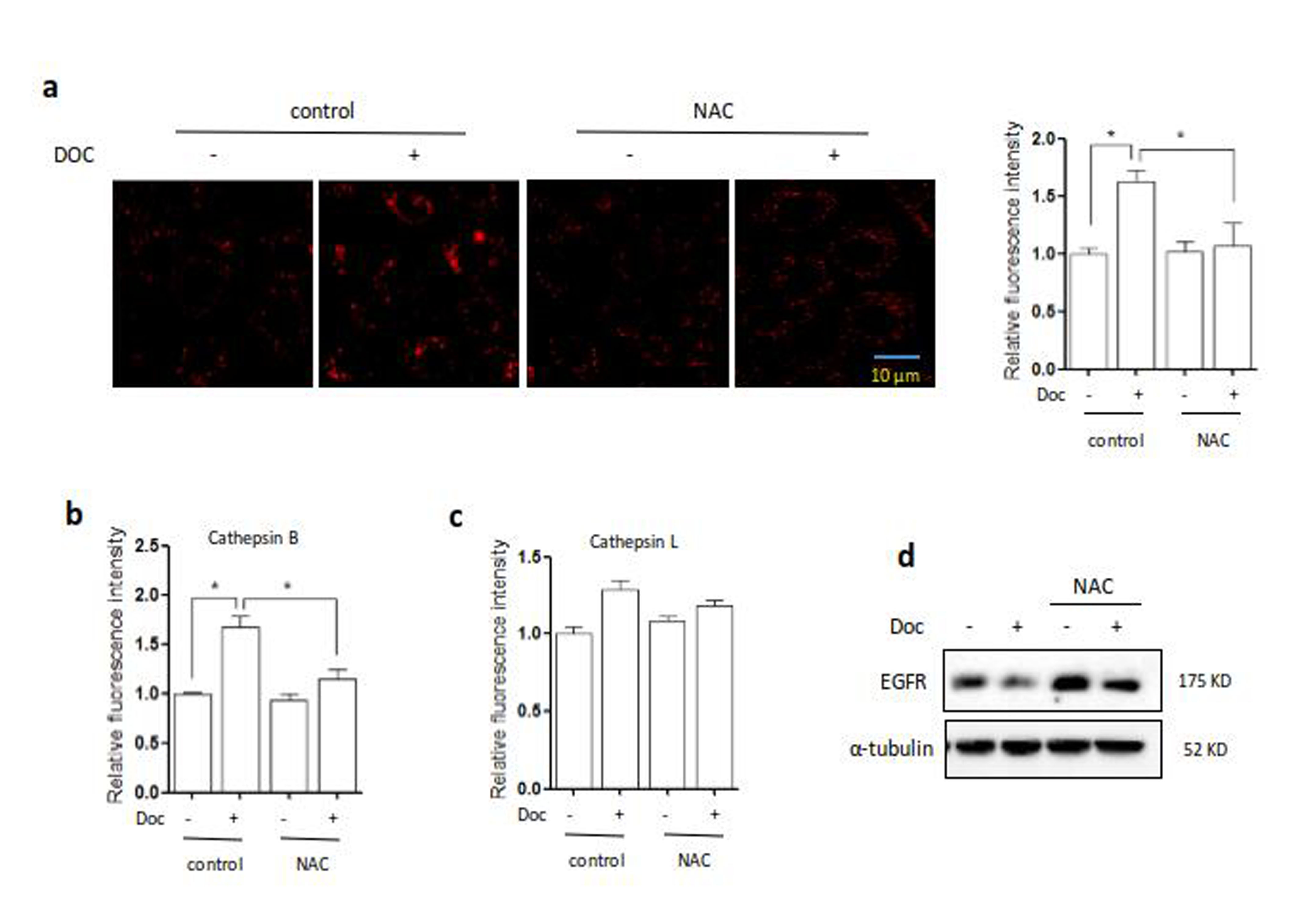

Supplement: Supplementary file 3 — supplementary Figure 3 [file 41419_2018_571_MOESM3_ESM.jpg]

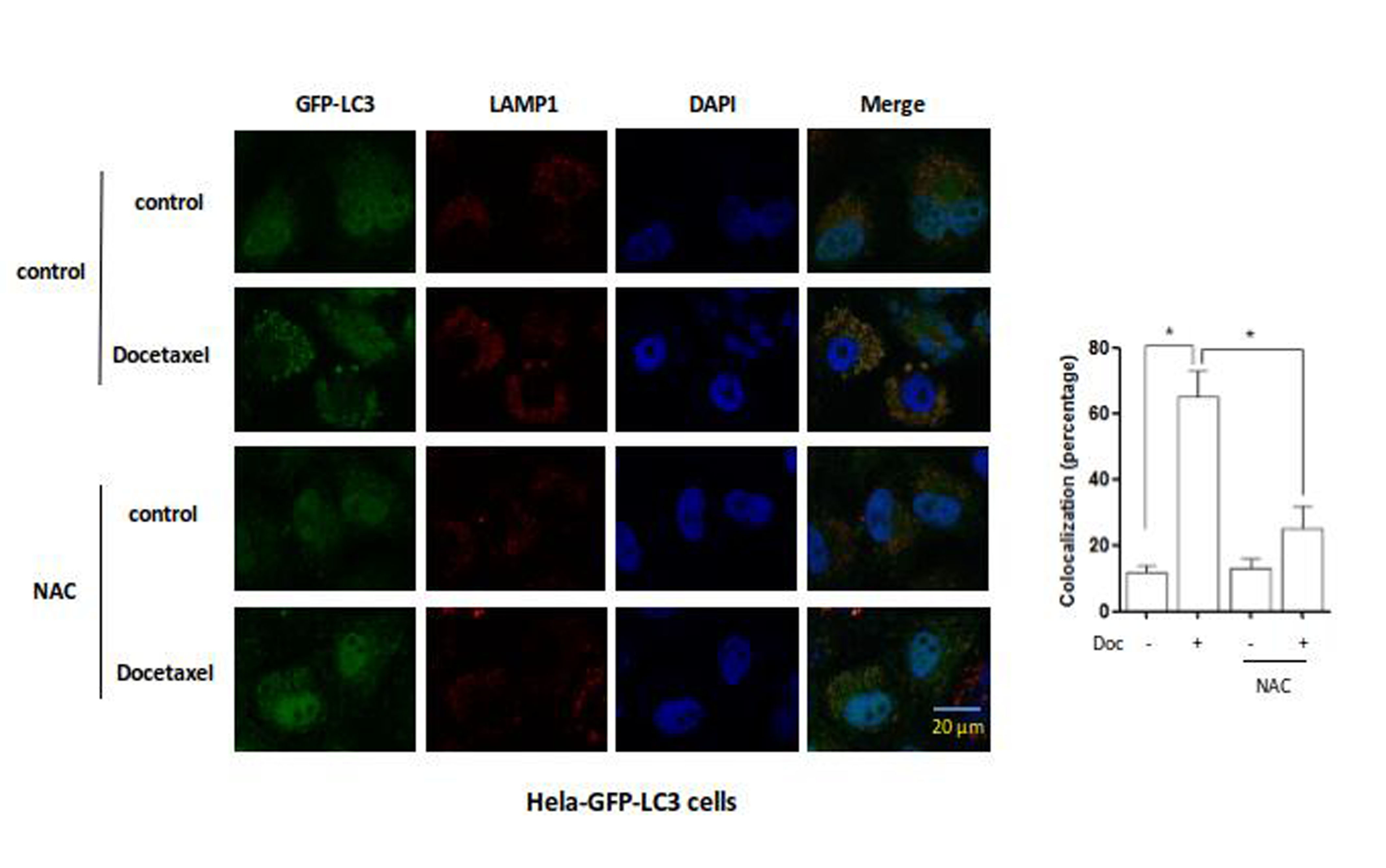

Supplement: Supplementary file 4 — supplementary Figure 4 [file 41419_2018_571_MOESM4_ESM.jpg]

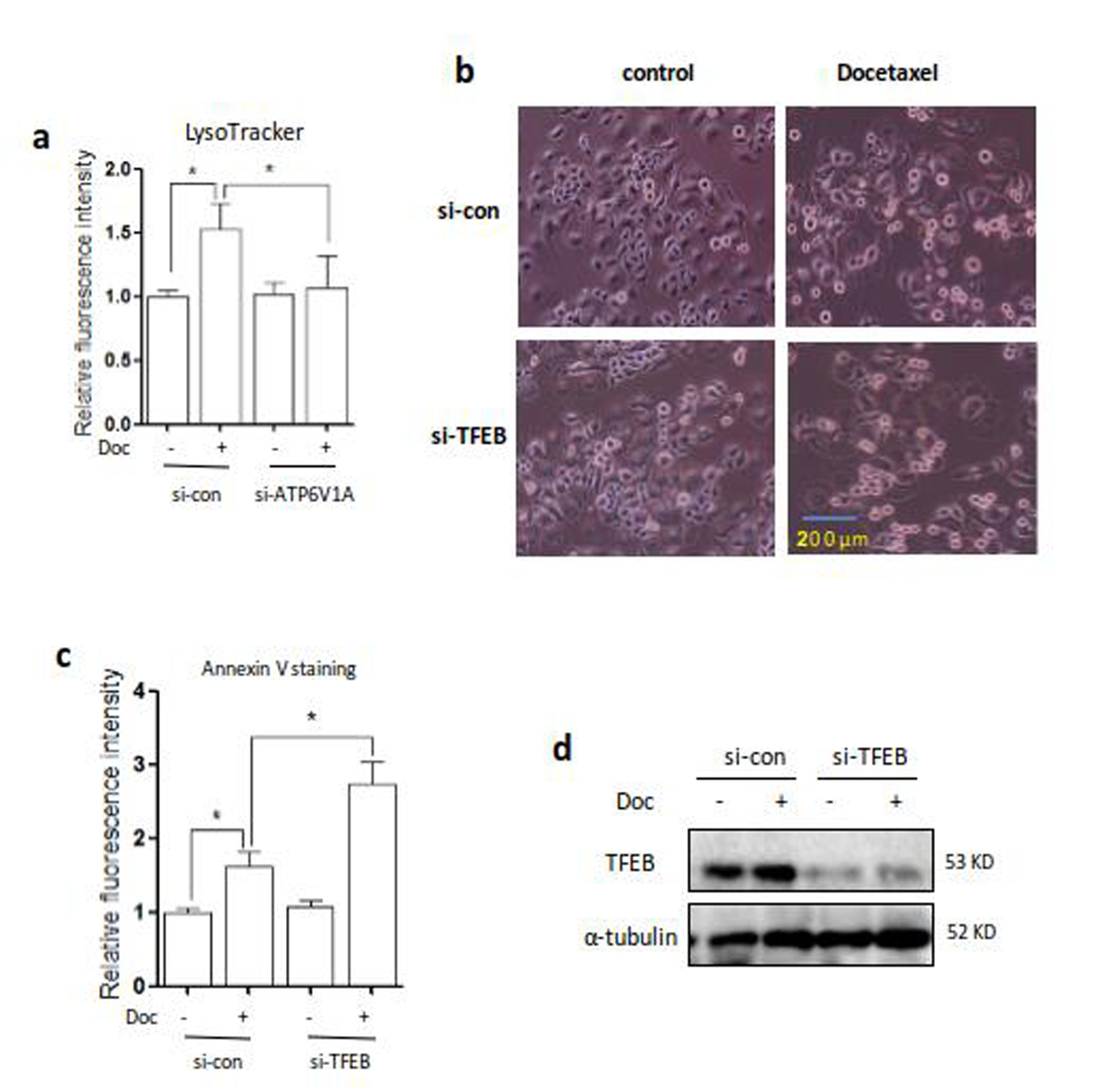

Supplement: Supplementary file 5 — supplementary Figure 5 [file 41419_2018_571_MOESM5_ESM.jpg]
