# Supplementary material for: Docetaxel enhances lysosomal function through TFEB activation
Source: Cell Death Dis. 2018 May 23;9(6):614. doi: 10.1038/s41419-018-0571-4 (PMC5966422; doi:10.1038/s41419-018-0571-4)
Supplement: Supplementary file 6 — supplementary materials [file 41419_2018_571_MOESM6_ESM.docx]

**Materials and Methods**

**Reagents and antibodies**

The antibodies used in our experiments included: ATP6V1A (Abcam, ab199326), EGF receptor (Cell Signaling Technology, 4267), LC3 (microtubule-associated protein 1 light chain 3)(Sigma, L7543), α-tubulin (Sigma, T6199), Lamin AC (Cell Signaling Technology,2032), LAMP1 (Cell Signaling Technology, 9091), SQSTM1 (Sigma, SAB1406748), TFEB (Bethyl Laboratories, A303-673A).

The chemicals used in our experiments were: Annexin V Pacific Blue™ conjugate (Thermo Fisher Scientific, A35122), acridine orange (AO) (Immunochemistry Technologies, LLC, 6130), docetaxel (Sigma, 01885), chloroquine (CQ) (PubChem, 2719), lysoTracker Red DND-99 (Invitrogen, L7528), lysoSensor green DND-189 (Invitrogen, L7535), Magic Red^TM^ cathepsin B and L reagent with Acridine Orange (Immunochemistry Technologies, LLC, 937/938/6130), thapsigargin (Sigma, T9033).

**Cell culture**

AGS and BGC803 cells were obtained from ATCC (AmericanType Culture Collection). Hela cells stably expressing GFP-LC3 were kindly provided by Dr. N. Mizushima (Tokyo Medical and Dental University, Japan). The tfLC3 stably transfected L929 cells were from Prof. Shen Hanming’s lab (National University of Singapore, Singapore). Cells were maintained in DMEM (Sigma, D1152) containing 10% fetal bovine serum (HyClone, SV30160.03) in a 5% CO_2_ atmosphere at 37°C.

**Acridine orange (AO) staining**

AO is a lysosomotropic weak base and a concentration dependent meta-chromatic fluorophore. Cell were stained with AO at a concentration of 5 μg/ml for 15 minutes and then washed with PBS. Fluorescence intensities of 10, 000 cells per sample were measured by flow cytometry using the FACS cytometer (BD Biosciences).

**Estimation of intralysosomal pH using lysoTracker**

The intralysosomal pH was estimated using LysoTracker, following manufacturer’s instructions. The fluorescence intensity was observed under fluorescence microscope (Nikon ECLIPSE) and representative cells were selected and photographed.

**Cathepsin B and L activity assay**

Following an earlier report ^1^, cells were cultured in 12-well plates and treated as indicated. After treatments, cells were further loaded with Magic Red cathepsin B or L reagent for 15 minutes. Fluorescence intensities of 10, 000 cells per sample were measured by flow cytometry using the FACS cytometer (BD Biosciences).

**Measurement of cellular fluorescence using fluorescence microscopy**

Cells were seeded onto glass slides. After the designated treatments, cells were first fixed with 4% paraformaldehyde and permeabilized by 0.25% Triton X-100. And then cells were incubated with SQSTM1 or LAMP1 antibodies, respectively. After incubation in fluorochrome-conjugated secondary antibody, cells fluorescence intensity was observed under fluorescence microscope (Nikon ECLIPSE) and representative cells were selected and photographed.

**Luciferase assay**

As described before ^23^, the luciferase vector was transiently transfected into AGS cells using Lipofectamine 2000 transfection reagent (Invitrogen, 11668) according to the manufacturer’s protocols. After the designated treatments, the luciferase activity was measured using a Dual-Luciferase Reporter Assay System (Promega, E1960) based on the protocol provided by the manufacturer.

**Cell fractions preparation**

AGS cells were treated with docetaxel at different time points. After that, nuclear and cytosolic extracts were then prepared with NE-PER^®^ nuclear and cytoplasmic extraction reagents (Pierce, 78833) according to the manufacturer’s protocol.

**Small interfering RNA (siRNA) and transient transfection**

The scrambled RNAi oligonucleotides and siRNAs targeting TFEB (Dharmacon, 21425) or ATP6V1A ([GenePharma](http://www.genepharma.com/about.php_blank), Shanghai) were transfected into AGS cells using the DharmaFECT 4 Transfection Reagent (Dharmacon, T-2001-02) according to the manufacturer’s protocol.

**Western blotting**

Cells were lysed in Laemmli SDS buffer (62.5 mM Tris, pH6.8, 25% glycerol, 2% SDS, phosphatase inhibitor and proteinase inhibitor cocktail). An equal amount of protein was resolved by SDS-PAGE and transferred onto PVDF membrane. After blocking with 5% nonfat milk, the membrane was probed with designated primary and secondary antibodies, developed with the enhanced chemiluminescence method and visualized with the ChemiDoc MP (BIO-RAD).

**Reverse transcription and quantitative Real-time PCR**

RNA was extracted with the RNeasy kit (Qiagen, 217004). 1 μg of total RNA was used for a reverse transcription reaction using High Capacity cDNA Reverse Transcription kit (Applied Biosystems, 4368814). Real-time PCR was performed to measure the mRNA expression levels using SsoFast Eva Green Supermix (Bio-Rad, 172-5201) and CFX96 Touch Real-time PCR Detection System (Bio-Rad). Glyceraldehyde-3-phosphatedehydrogenase (GAPDH) was used as an internal control of RNA integrity. Real-time PCR was performed in triplicate. The primers used for ATP6V1A, TFEB, UVRAG and GAPDH were based on the previous report ^21^.

**Detection of viable and dead cells**

Cell death was determined quantitatively and qualitatively using the following two assays, which are (i) morphological changes under phase-contrast microscopy; (ii) Annexin V staining coupled with flow cytometry. For Annexin V staining, the medium in each well was collected and cells were harvested with trypsin after treatments. Then, cell pellets obtained were resuspended in 1× Binding buffer containing 5 μL of Annexin V and incubated for 15 minutes at room temperature. Ten thousand cells from each sample were analyzed with FACS Calibur flow cytometry (BD Bioscience, San Jose, CA) using CellQuest software.

**Statistical analysis**

All western blotting and image data presented are representatives from at least 3 independent experiments. The numeric data are presented as means ± SD from 2-3 independent experiments (each in duplicates or triplicates) and analyzed using Student’s t-test.
