# Supplementary material for: Docetaxel enhances lysosomal function through TFEB activation
Source: Cell Death Dis. 2018 May 23;9(6):614. doi: 10.1038/s41419-018-0571-4 (PMC5966422; doi:10.1038/s41419-018-0571-4)
Supplement: Supplementary file 7 — Supplementary figure legends [file 41419_2018_571_MOESM7_ESM.docx]

**Supplementary Figure legends**

**SF. 1 Activation of lysosomal function in cells treated with docetaxel. a** BGC803 cells were treated with docetaxel (10 nM, 12 hours). After treatment, cells were stained with LysoTracker Red (50 nM) for 15 min and cell fluorescence was examined using confocal microscope (left panel) and quantified by flow cytometry (right panel). * *P* < 0.05 **b** and **c** AGS cells were treated with docetaxel (10 nM) for 6 or 12 hours as indicated. Magic Red^TM^ for cathepsin B or L reagent was loaded for 15 min. Fluorescence intensity of 10, 000 cells per sample was measured by flow cytometry. * *P* < 0.05

**SF. 2 Docetaxel activates TFEB function in AGS cells. a** AGS cells were treated with docetaxel (10 nM) with or without NAC(5 mM) for 12 hours. Cells were harvested for western blotting to determine TFEB level. α-tubulin was used as loading control.

**SF. 3 Docetaxel enhances lysosomal function through ROS generation. a** AGS cells were treated with docetaxel (10 nM) for 12 hours with or without NAC (5 mM). Cells were then stained with lysoTracker Red DND-99 (50 nM) for 15 min and cells fluorescence intensity was observed under fluorescence microscope (scale bar 10 μm, left panel) and quantified by flow cytometry (right panel). * *P* < 0.05 **b** and **c**, as described in a, cells were then loaded with Magic Red^TM^ for cathepsinB or L reagent for 15 min. Fluorescence intensity of 10, 000 cells per sample was measured by flow cytometry.* *P* < 0.05 **d** as in a, AGS cells were harvested and EGFR was analysed using western blotting. α-tubulin was used as loading control.

**SF 4. ROS scavenging disrupts the fusion of autophagosome and lysosome by docetaxel.** Hela cells stably expressing GFP-LC3 were treated with docetaxel (10 nM, 12 hours) with or without NAC (5 mM). After immunostaining with LAMP1 (Alexa Fluor 594, red), cells were examined by confocal microscopy (scale bar 20 μm). The colocalization of GFP-LC3 puncta and LAMP1 was calculated and statistically analysed. * *P* < 0.05

**SF 5. Lysosomal inhibition sensitizes docetaxel-induced cell death. a** AGS cells were transfected with scrambled or ATP6V1A siRNA for 48 hours and then treated with docetaxel (10 nM, 12 hours). After loading with LysoTracer Red, cell fluorescence was quantified by flow cytometry. * *P* < 0.05 **b** and **c** AGS cells were transfected with scrambled or TFEB siRNA for 48 hours and then treated with docetaxel (25 nM; 24 hours). Changes in AGS cell morphology following each treatment were captured with an inverted microscope (scale bar 200 μm). Cell pellets were subsequently collected and quantification of cell death was performed using Annexin V staining. Statistical significance (* *P*< 0.05) is indicated in the bar chart. **d** as in b, cells were harvested for western blotting to determine TFEB level. α-tubulin was used as loading control.
